# Global trends in research on cervicogenic headache: a bibliometric analysis

**DOI:** 10.3389/fneur.2023.1169477

**Published:** 2023-04-20

**Authors:** Yu Xu, Ying Gao, Lin Jiang, Lunhui Wu, Jing Yin, Zhijun Yang, Youkang Dong

**Affiliations:** ^1^Second Clinical Medical College, Yunnan University of Chinese Medicine, Kunming, Yunnan, China; ^2^Department of Tuina, The First Affiliated Hospital of Yunnan University of Chinese Medicine, Kunming, Yunnan, China; ^3^Department of Rehabilitation, Lincang Municipal Hospital of Chinese Medicine, Lincang, Yunnan, China

**Keywords:** cervicogenic headache, bibliometrix, visualization, network analysis, research frontiers, Web of Science

## Abstract

**Background:**

There has been a marked increase in cervicogenic headaches in recent years, significantly affecting sufferers’ daily lives and work. While several treatments exist for this type of headache, their long-term effects could be improved, and additional data from large clinical samples are needed. This study aims to systematically examine the current state of research in cervicogenic headaches through a bibliometric analysis, identify areas of current interest, and provide insight into potential future research directions.

**Methods:**

This article examines research trends in the field of cervicogenic headache through a bibliometric analysis of scholarly articles in the field of cervicogenic headache over the past four decades. The bibliometric analysis method employed included searching the Web of Science database using topics related to cervicogenic headaches. Inclusion criteria were limited to articles and review papers on cervicogenic headaches published between 1982 and 2022. The retrieved dataset was then analyzed using R software and VOSviewer to identify the major research areas, countries and institutions, the most influential authors, journals and keywords, co-citations in the literature, and co-authorship networks.

**Results:**

This study analyzed 866 articles published between 1982 and 2022, involving 2,688 authors and generating 1,499 unique author keywords. Neuroscience and neurology were the primary focus, with participation from 47 countries, primarily led by the United States, which has the most published articles (*n* = 207), connections (*n* = 29), and citations (*n* = 5,238). In the cervicogenic headache study, which involved 602 institutions, the University of Queensland received the most significant number of citations (*n* = 876), and Cephalalgia was the journal with the most published articles and received the most local citations (*n* = 82) and highest growth (*n* = 36). Two hundred sixty-nine journals have published articles on cervicogenic headaches. Among researchers studying cervicogenic headache, Sjaastad O had the most published articles (*n* = 51) and citations (*n* = 22). The most commonly occurring keyword was “cervicogenic headache.” Except for the fourth most impactful paper, as determined by the Local Citation Score, which analyzed clinical treatments, all the top documents emphasized investigating the diagnostic mechanisms of cervicogenic headache. The most commonly occurring keyword was “cervicogenic headache.”

**Conclusion:**

This study used bibliometric analysis to provide a comprehensive overview of the current research on cervicogenic headaches. The findings highlight several areas of research interest, including the need for further investigation into the diagnosis and treatment of cervicogenic headaches, the impact of lifestyle factors on cervicogenic headaches, and the development of new interventions to improve patient outcomes. By identifying these gaps in the literature, this study provides a foundation for guiding future research to improve the diagnosis and treatment of cervicogenic headaches.

## 1. Introduction

Cervicogenic Headache (CEH) is a headache caused by neck or paravertebral soft tissue lesions (1). This headache can be episodic or chronic, characterized by recurrent, one-sided headaches with accompanying neck pain and stiffness (1–3). This headache is thought to account for 15% to 20% of all headaches (4). Studies have shown that CEH is prevalent among individuals over 50 with headaches, with estimates ranging from 0.4% to 42% (5). Lifestyle changes have influenced the incidence of CEH in recent years. Prolonged sitting in a poor posture can increase the risk of CEH (6, 7), because prolonged sitting can cause muscular imbalances, joint stiffness, and trigger points, leading to increased tension and pain in the neck muscles (6, 8). Repetitive neck movements, such as those performed during manual labor or computer work, have been associated with an increased risk of CEH (9, 10) because they can cause strain on the neck muscles and joints, leading to increased tension and pain. CEH can significantly impact the daily life and work of sufferers, leading to decreased productivity and quality of life. While various treatments are available for CEH, their efficacy is limited, and the long-term clinical effectiveness and availability of extensive sample data require improvement (11).

“Bibliometrics,” as first introduced by Pritchard (12), constitutes a formidable technique for analyzing the advancement of scientific research. It enables quantifying information extracted from online science citation databases on a particular topic, including the distribution of authors, publications, and research institutions within the field. Furthermore, bibliometrics can identify important literature in a research area, providing relevant keywords, information about institutions, connections to countries, and a visual representation of the distribution of the literature in the form of a knowledge map. This information can identify current and future trends in research topics and guide future research directions (13). For example, a study by Zhao et al. (14) used bibliometric analysis to identify the most influential articles and authors in migraine research. Similarly, a study by Downes et al. (15) used bibliometric analysis to examine the trends and therapeutic applications of vagal nerve stimulation research. These studies demonstrate the value of bibliometric analysis in understanding the structure and evolution of headache research and inform the design of future studies and interventions, ultimately benefiting patients. As such, we utilize bibliometric analysis to identify the most influential authors, publications, articles, countries, and institutions in headaches, examine current and future research trends of CEH, and provide insights into potential directions for future research.

## 2. Methods

### 2.1. Literature retrieval strategy

Studies have shown that the Web of Science (WOS) database is the most suitable bibliometric analysis database (16). This paper aims to explore the literature on CEH using bibliometric analysis. We used the WOS Core Collection All database to retrieve 1,031 documents (Query date is 1 January 2023) related to CEH using the search formula TS = (“Cervicogenic headache*” OR “Cervical headache*”). We exported all records as “Plain text files” with “Full Records and Cited References.” The purpose of this search is to provide valuable insights into the structure and evolution of the field of CEH research, including identifying key authors, institutions, and research trends.

### 2.2. Bibliometric analysis

**Figure 1:**
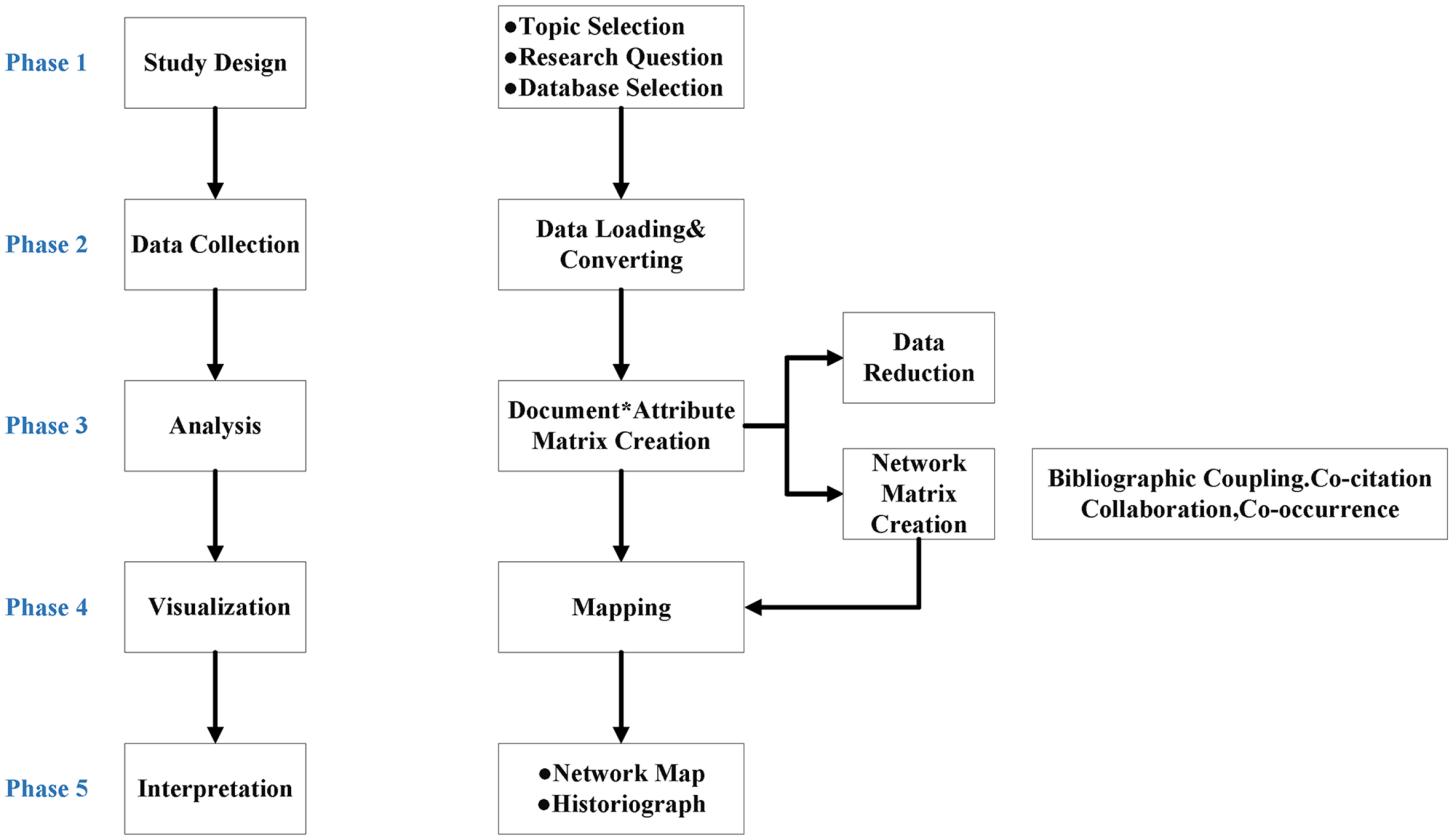
Schematic depicting bibliometric analysis methodologies.

In their publication, Aria and Cuccurullo et al. (17) detailed a bibliometric analysis utilizing a five-step methodology: study design, data collection, analysis, visualization, and interpretation (18–20). Figure 1 illustrates a schematic representation of this approach. The study design phase involved selecting the topic of CEH and using the WOS Core Collection All database as the data source. We conducted the literature search during the data collection phase. To maintain the validity of the research and ensure reliable scientific communication (20), we use document-type filters on WOS and only include articles and review papers. We imported all the records using Biblioshiny Web, then converted them to Bibliometrix Rdata and Excel for further analysis. We created a matrix of all the papers using R 4.2.2 and used different data analysis and visualization tools. Specifically, we used the following tools:

1. biblioshiny: we used the biblioshiny package to open Biblioshiny Web, clean, preprocess the data, and generate descriptive statistics.
2. tidyverse (ggplot2): we used tidyverse, belong the ggplot2 package, to create high-quality graphics and visualizations.
3. VOSviewer 1.6.18: we used VOSviewer to generate concept maps, co-citation networks, and alternative graphs for further analysis.

Bradford’s law is a bibliometric principle that explains how scientific literature is distributed across various publications in a given field. It states that a small group of highly productive publications, known as ‘core’ publications, publish most articles in that field. The remaining articles are published in a larger number of less productive publications, called ‘dispersed’ publications. This principle is based on the observation that the number of articles published in a given publication is inversely proportional to its rank when publications are ranked by decreasing productivity (21). Bradford’s law was then employed to examine the distribution of journals and pinpoint influential sources. The data analysis and visualization results, including insights obtained from Bradford’s law, will be discussed in the latter half of the paper.

The authors conducted this bibliometric study alone. We searched the WOS database using specific search terms to identify the relevant articles and limited our results to articles published between 1982 and 2022. We then extracted the necessary data, such as author affiliations, publication year, and citation counts, and conducted a detailed analysis of the results.

## 3. Results and discussion

The preliminary findings of the bibliometric analysis present a comprehensive overview of the statistics in the field, and we ended up including only 866 papers published between 1982 and 2022. Subsequently, we analyze the aspects such as authors, journals, themes, keywords, and countries represented in the relevant literature to delve further into the research.

### 3.1. Descriptive bibliometric analysis

**Figure 2:**
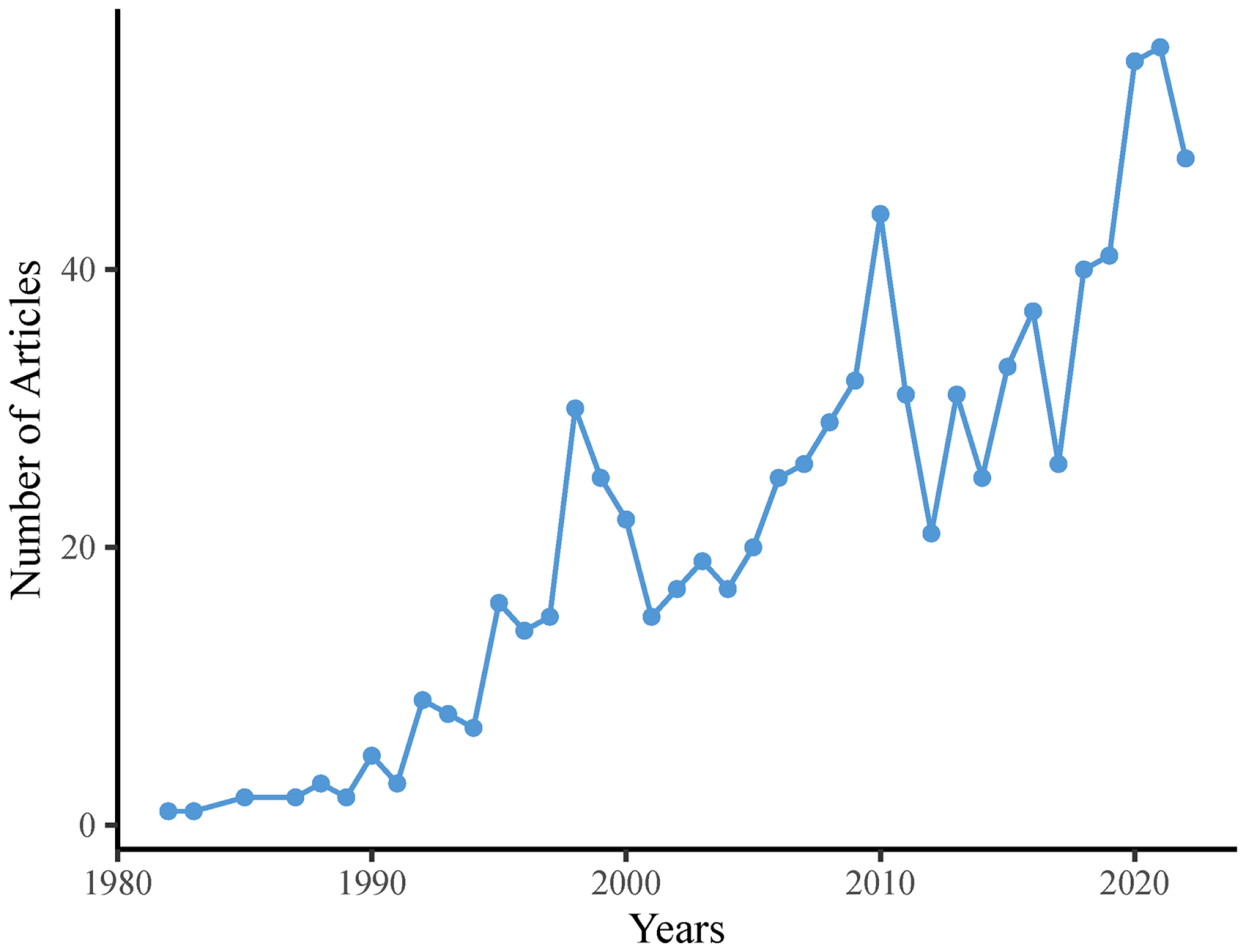
Scientific production of CEH-related literature between 1982 and 2022.

During the study period, Figure 2 depicts the overall scientific findings. The year 1982 marked the debut of CEH research with the publication of “The True Cervical Headache” (22) in the WOS database, with only a single paper that year. CEH-related paper publication increased after 1982, with some interruptions in 1984 and 1986. The trend fluctuated over the four decades from 1982 to 2022. However, CEH-related studies increased significantly after 2017 and reached 56 in 2021. Table 1 summarizes the key statistics of the 866 CEH-related articles published in the WOS Core Collection All database between 1982 and 2022. Over 40 years, the average number of CEH research papers published annually is 22, with average of 24.58citations each paper. These studies involved 2,688 authors, including 68 single-author documents. Additionally, the CEH research papers generated 1,499 author keywords.

**Table 1:** Primary statistics of CEH-related literature derived from bibliometric analysis.

Main information	Description	Value
Documents	Total number of documents	866
Sources	The frequency distribution of sources as journals, books, etc.	269
Timespan	Years of publication	1982–2022
References	Total number of references	18,019
Author’s keywords (DE)	Total number of author’s keywords	1,499
Keywords plus (ID)	Total number of phrases that frequently appear in the title of an article’s references	1,440
Authors	Total number of authors	2,688
Authors appearances	The authors’ frequency distribution	3,776
Authors of single-authored documents	The number of single authors per articles	68
Authors per document	Average number of authors in each document	0.322
Co-Authors per documents	Average number of co-authors in each document	4.36
Average citations per documents	Average number of citations in each document	24.58

### 3.2. Research areas

**Figure 3:**
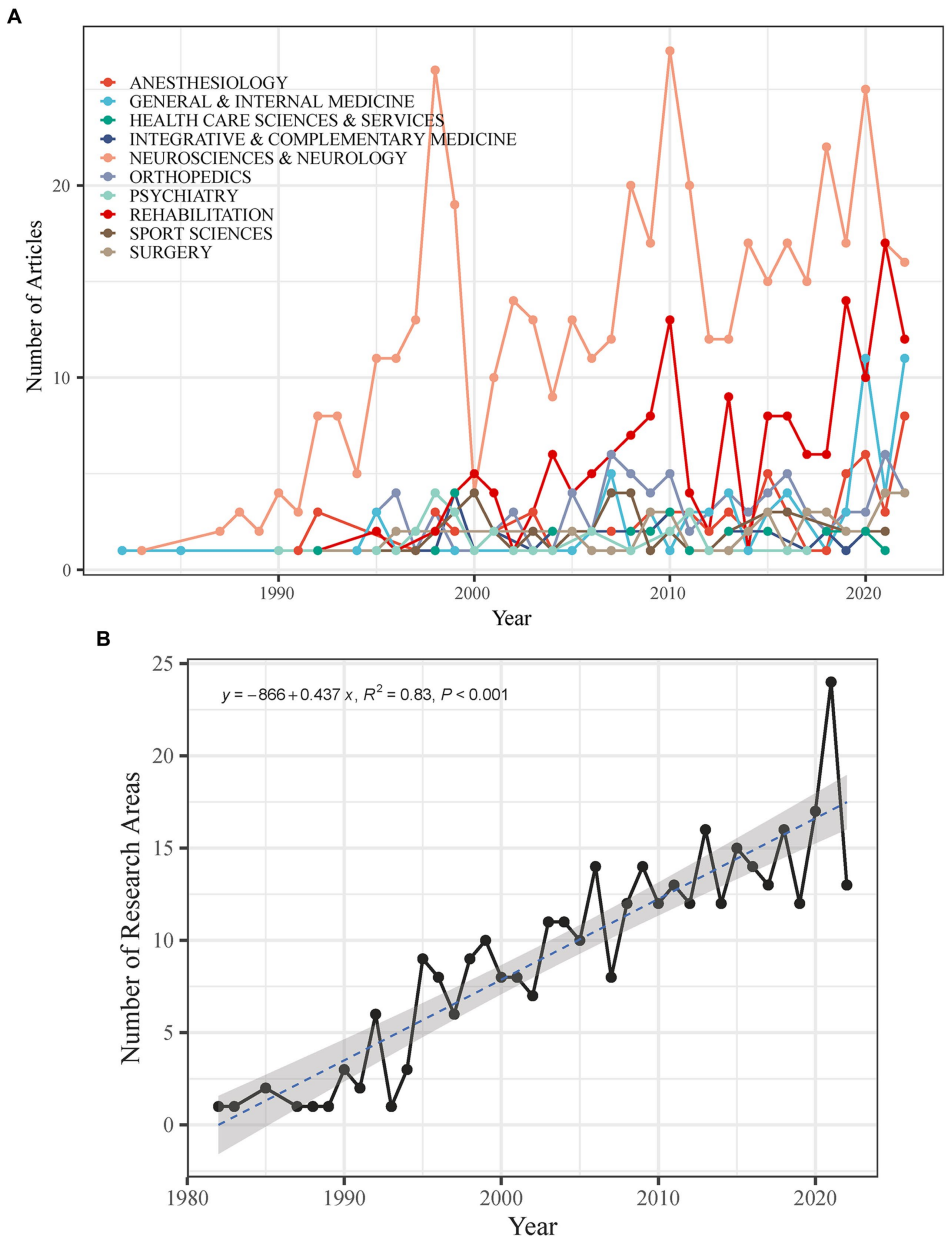
**(A)** Number of research areas in the CEH-related literature. **(B)** Temporal evolution of the top 10 most fecund research areas in the CEH-related literature.

According to Clarivate Analytics (23), the WOS research areas were used to categorize research papers. Each paper in the WOS database can be allocated to at least one research area. The research shows that the coverage of CEH research fields has expanded from a single field in 1982 to 24 fields in 2021. These were the top 10 most productive research areas in CEH (Figure 3A): Neuroscience and Neurology, Rehabilitation, Orthopedics, General and Internal Medicine, Anesthesiology, Sport Sciences, Surgery, Integrative and Complementary Medicine, Health Care Sciences and Services, and Psychiatry. They made up 87.46% of the total CEH-related publications. Figure 3B provides a visual representation of how the focus areas of CEH research have changed over the years. Between 1982 and 2022, the leading research area was Neuroscience and Neurology, reaching its peak in terms of publication numbers in 2010.

### 3.3. Research countries and institutions

**Figure 4:**
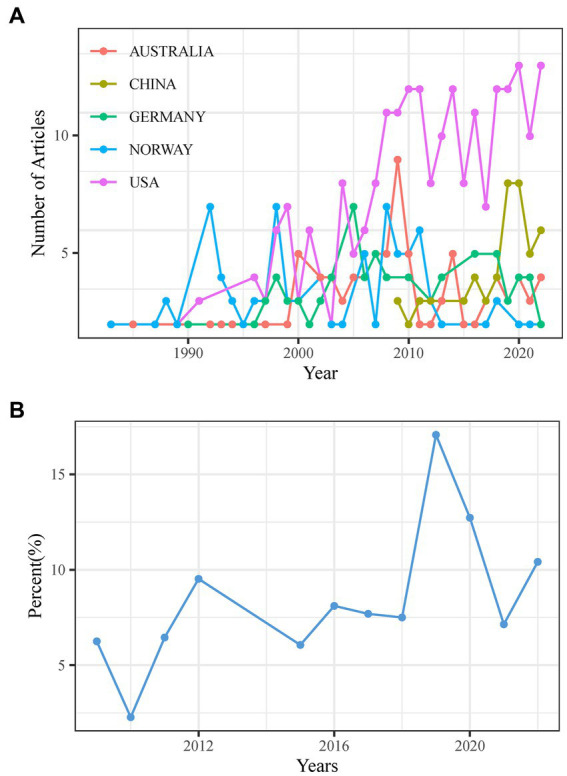
**(A)** Top 5 countries in annual scientific production. **(B)** Annual proportion of scientific production in China.

**Figure 5:**
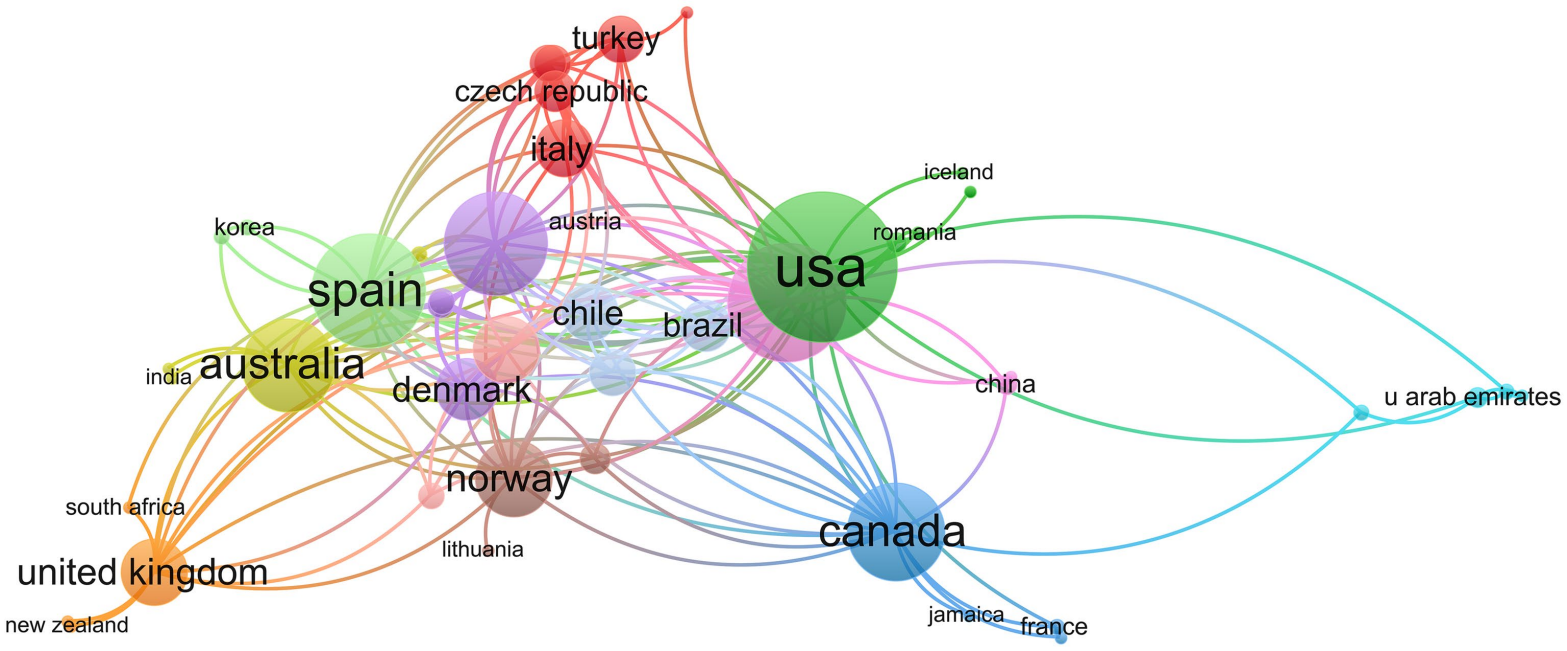
Map of inter-nation research collaboration.

The findings demonstrate that 47 countries currently invest in CEH research. The United States of America (United States; *n* = 207), Norway (*n* = 71), Australia (*n* = 70), Germany (*n* = 58), and China (*n* = 40) are the top five nations regarding scientific output. After 2005, the number of publications from the United States experienced a steep and sustained increase and eventually came to dominate (Figure 4A). China’s contribution to CEH scientific production demonstrates fluctuations between high and low levels, reaching a peak of 19.57 in 2019 (Figure 4B). In addition, to quantifying the production of scientific results, we can use country collaboration maps to gauge a country’s research capacity. A global network of collaborations is represented by Figure 5 and reveals that the United States (*n* = 29) holds the most connections, followed by Germany (*n* = 23), Spain (*n* = 22), and the Netherlands (*n* = 20). Different countries exhibit fewer CEH research collaborations, with at most 20 connections.

**Figure 6:**
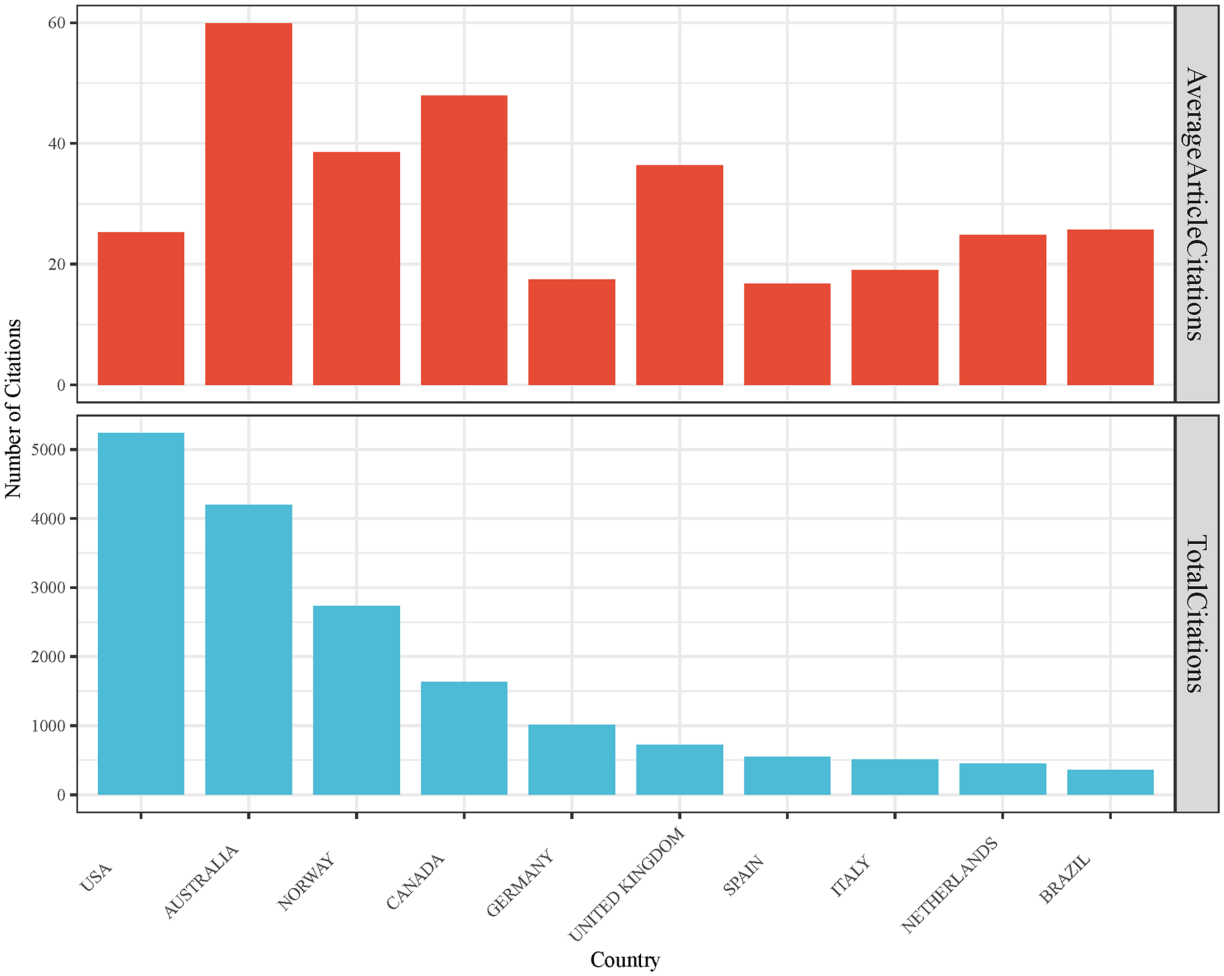
Total and average citations for the top 10 most frequently cited countries.

After analyzing the data, we determined the overall paper count for each country. Additionally, we assessed each article’s total number of citations and calculated the average number of citations to extract the top 10 countries (Figure 6). The United States was by far the most frequently cited country (*n* = 5,238), followed by Australia (*n* = 4,195), Norway (*n* = 2,734), Canada (*n* = 1,630), Germany (*n* = 1,011), the United Kingdom (*n* = 727), Spain (*n* = 552), Italy (*n* = 513), the Netherlands (*n* = 447), and Brazil (*n* = 360). Regarding average article citations, the top 10 countries demonstrate less variation. Australia holds the highest average citation count (*n* = 59.93), followed by Canada (*n* = 47.94), Norway (*n* = 38.51), the United Kingdom (*n* = 36.35), Brazil (*n* = 25.71), the United States (*n* = 25.30), the Netherlands (*n* = 24.83), Italy (*n* = 19.00), Germany (*n* = 17.43), and Spain (*n* = 16.73). These results highlight the dominance of the United States in CEH-related research.

A total of 602 institutions worldwide are involved in CEH research. Each institution’s significance was measured based on the number of citations received for their published papers. The leading 10 institutions with the most citations for their papers were considered the most impactful. The study found that the influence of papers from different institutions varied significantly. The University of Queensland (*n* = 876) had the highest number of citations, followed by McMaster University (*n* = 662), NTNU (*n* = 462), Rey Juan Carlos University (*n* = 292), the University of Washington (*n* = 280), Newcastle Bone and Joint Institute (*n* = 275), the University of South Australia (*n* = 264), Neurology Institute (*n* = 245), Women’s College Hospital (*n* = 226), Newcastle University (*n* = 209; Table 2). While Australia has a low number of publications, it has a significant quantity of citations, which significantly impacts CEH research.

**Table 2:** Top 10 institutions based on total citations in CEH-related research.

Institution	Country	Total number of articles	Total number of citations
University Queensland	Australia	876	5
McMaster University	Canada	662	6
NTNU	Norway	462	6
University Rey Juan Carlos	Spain	292	8
University Washington	United States	280	2
Newcastle Bone and Joint Institute	Australia	275	2
University of South Australia	Australia	264	1
Neurol Institution	United Kingdom	245	1
Women’s College Hospital	Canada	226	2
University Newcastle	Australia	209	4

NTNU, Norwegian University of Science and Technology.

### 3.4. Leading source journals

**Figure 7:**
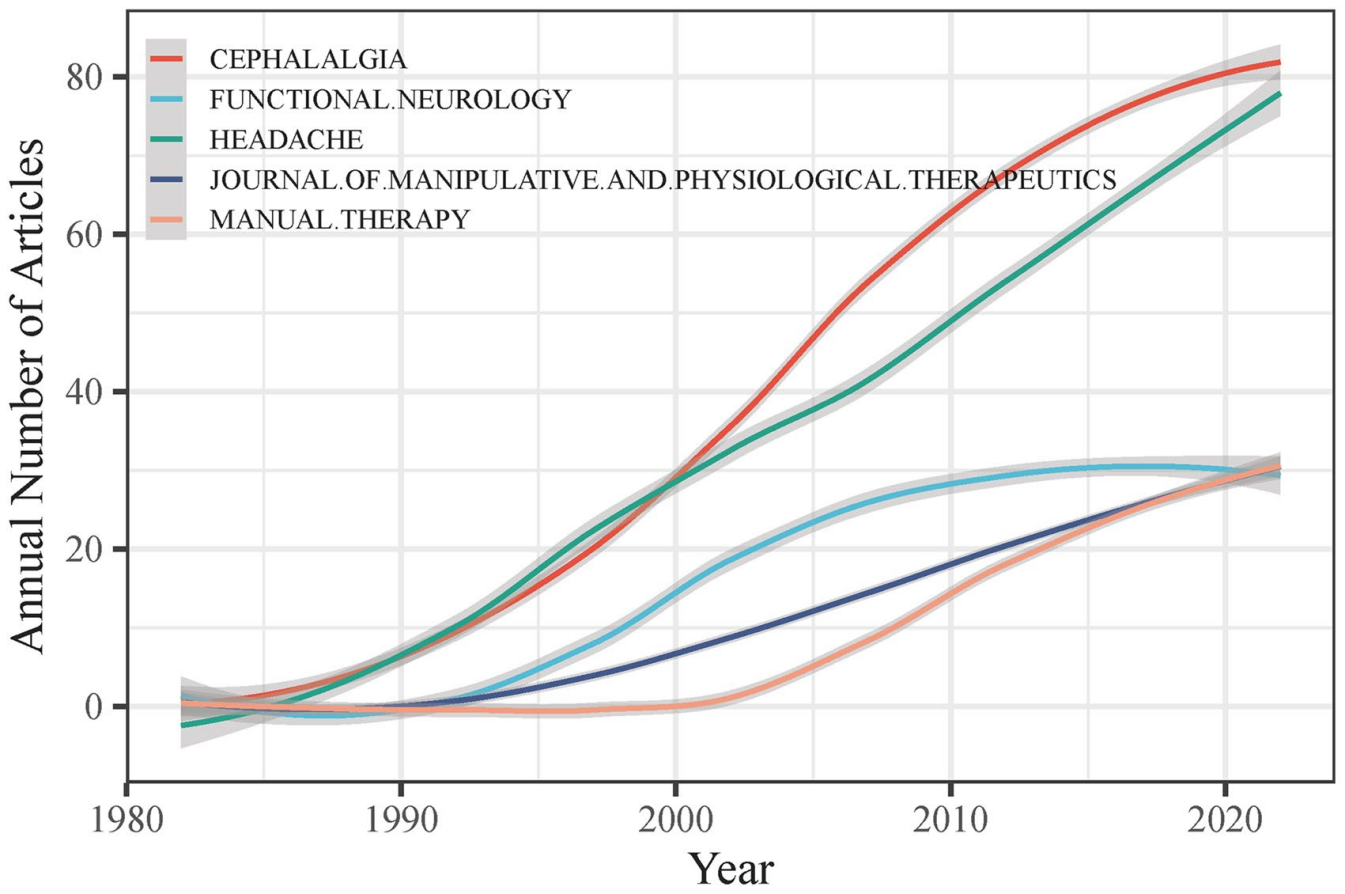
Temporal analysis of CEH-related study publication sources.

CEH-related studies have been reported in 269 journals. After analyzing the distribution of CEH research papers among the primary sources, we found that the top 5 journals published 219 papers. However, 172 journals published only one CEH paper, and 251 journals posted at most 10 articles. Figure 7 shows that the top 5 journals with the most published papers were Cephalalgia (*n* = 82), Headache (*n* = 77), Functional Neurology (*n* = 30), Journal of Manipulative and Physiological Therapeutics (*n* = 29), Manual Therapy (*n* = 28). Cephalalgia also had the highest growth in published papers and total local citations (Table 3). As determined by Bradford’s law, the source journals for CEH research papers are scattered, and the top 10 journals are chosen based on the number of local citations. Journals marked with an asterisk are considered essential sources in the study of CEH based on Bradford’s Law, including Cephalalgia, Headache, Spine, Journal of Manipulative and Physiological Therapeutics and Manual Therapy, Journal of Headache and Pain, Journal of Orthopaedic and Sports Physical Therapy, Functional Neurology. These journals have significantly influenced research in CEH.

**Table 3:** Ranking of the top 10 journals with the most local citations for CEH-related research.

Sources	Total number of local citations	Number of articles	IF	H index
Cephalalgia^*^	3,624	82	6.075	36
Headache^*^	2,883	77	5.311	34
Spine^*^	1,947	14	3.269	11
Pain	1,238	7	7.926	7
Journal of Manipulative and Physiological Therapeutics^*^	969	29	1.3	17
Manual Therapy^*^	833	28	2.622	22
Neurology	577	1	12.258	1
Journal of Headache and Pain^*^	574	14	8.588	12
Journal Of Orthopaedic and Sports Physical Therapy^*^	451	16	6.276	13
Functional Neurology^*^	409	30	0.51	12

X*, the core journal of CEH research as defined by Bradford Law; IF, impact factor in 2021.

### 3.5. Most influential authors

The H-index is a widely recognized metric for assessing the scientific impact of a researcher based on the number of citations received for their published papers (24). We calculated the H-index for all authors who had published papers on CEH research in our dataset of 866 papers, regardless of their position in the authorship list. We identified 10 authors with the highest H-index scores, including Sjaastad O. (*n* = 22), Jull G. (*n* = 15), Goldsmith C. H. (*n* = 14), Bovim G. (*n* = 12), Fernandez-De-Las-Penas C. (*n* = 12), Gross A. (*n* = 12), Bronfort G. (*n* = 11), Burnie S. J. (*n* = 11), Fredriksen T. A. (*n* = 11), and Hall T. (*n* = 11; Table 4). Sjaastad O, the earlier recorded author in the WOS database for CEH research, was found to have the most significant number of published articles and citations. Three of the top 10 most influential researchers are from Norway, three are from Canada, and the rest are from United States, Australia, and Spain. In total, the study analyzed 866 papers, with 2,688 authors, with 68 authors having published single-authored literature. Co-authors per paper averaged 4.36, pointing to the tendency for CEH research to involve multi-author collaborations.

**Table 4:** Top 10 most influential authors based on the H-index.

Author	H index	G index	Cited count	NP	PY_start	Country
Sjaastad O.	22	44	2,011	51	1983	Norway
Jull G.	15	23	1,800	23	1994	Australia
Goldsmith C.H.	14	16	1,181	16	2005	Canada
Bovim G.	12	12	776	12	1991	Norway
Fernandez-De-Las-Penas C.	12	18	432	18	2005	Spain
Gross A.	12	14	1,024	14	2005	Canada
Bronfort G.	11	11	1,118	11	2001	United States
Burnie S.J.	11	11	951	11	2009	Canada
Fredriksen T.A.	11	20	892	20	1987	Norway
Hall T.	11	17	646	17	2004	Australia

NP, number of scientific productions; PY_start, Year of first publication of articles.

### 3.6. Most influential papers

We organized the most influential papers based on their Local Citation Score (LCS) and Global Citation Score (GCS), which are measures of the citation impact of a paper in a given field and across all fields, respectively (25). We used the LCS and GCS to determine the significance of each paper in the field of CEH. Papers with high LCS and low GCS scores were considered highly significant in CEH, while papers with high GCS and low LCS scores were considered highly significant across all fields (26, 27) (Tables 5, 6).

**Table 5:** Top 10 papers with the highest local citation score.

Paper	DOI	Year	Local citation score	Global citation score
Sjaastad O, 1998, Headache	10.1046/j.1526-4610.1998.3806442.x	1998	240	308
Sjaastad O, 1990, Headache	10.1111/j.1526-4610.1990.hed3011725.x	1990	171	238
Sjaastad O, 1983, Cephalalgia	10.1046/j.1468-2982.1983.0304249.x	1983	142	189
Jull G, 2002, Spine	10.1097/00007632-200,209,010-00004	2002	124	447
Bogduk N, 2009, Lancet Neurol	10.1016/S1474-4422(09)70209-1	2009	117	188
Zito G, 2006, Manual Ther	10.1016/j.math.2005.04.007	2006	83	172
Bovim G, 1992, Pain	10.1016/0304-3.959(92)90007-X	1992	82	122
Sjaastad O, 2008, Acta Neurol Scand	10.1111/j.1600-0404.2007.00962.x	2008	78	95
Bovim G, 1992, Pain	10.1016/0304-3.959(92)90237-6	1992	77	99
Nilsson N, 1995, Spine	10.1097/00007632-199.509.000-00008	1995	73	96

DOI, Digital Object Identifier.

**Table 6:** Top 10 papers with the highest global citation score.

Paper	DOI	Year	Local citation score	Global citation score
Jull G, 2002, Spine	10.1097/00007632-200.209.010-00004	2002	124	447
Childs JD, 2008, J Orthop Sport Phys	10.2519/jospt.2008.0303	2008	12	398
Sjaastad O, 1998, Headache	10.1046/j.1526-4610.1998.3806442.x	1998	240	308
Watson DH, 1993, Cephalalgia	10.1046/j.1468-2982.1993.1304272.x	1993	60	264
Afridi SK, 2006, Pain	10.1016/j.pain.2006.01.016	2006	31	245
Jull GA, 2008, J Manip Physiol Ther	10.1016/j.jmpt.2008.08.003	2008	12	244
Sjaastad O, 1990, Headache	10.1111/j.1526-4610.1990.hed3011725.x	1990	171	238
Sterling M, 2003, Pain	10.1016/S0304-3959(02)00420-7	2003	7	236
Miller J, 2010, Manual Ther	10.1016/j.math.2010.02.007	2010	14	209
Jull G, 1999, Cephalalgia	10.1046/j.1468-2982.1999.1903179.x	1999	44	191

DOI, Digital Object Identifier.

The first and second most influential papers in LCS (ranked 3 and 7 for GCS) were published by the author SJAASTAD O in 1998 and 1990, respectively. The latter paper develops the diagnostic criteria for CEH, and the former adds the signs and symptoms of neck involvement. This former paper also distinguishes the diagnosis of CEH from other headaches and clarifies the pain mechanism involved. The diagnostic criteria presented in this former paper are more comprehensive and offer a more robust understanding of the condition (28, 29). These two papers by SJAASTAD O have made a significant contribution to the field of CEH and continue to be highly cited and influential.

The article “‘Cervicogenic’ headache. An hypothesis” by SJAASTAD O has been identified as the third most influential paper in the literature on CEH by the LCS. Although not ranking in the GCS top 10, this paper is considered seminal. The study aimed to determine the specificity of CEH and propose partial diagnostic criteria for the condition. The author identified specific criteria for CEH through the diagnostic treatment of a cohort of headache patients. The results of this study have been valuable for subsequent research in the field (30). In a subsequent study, ranked fourth in the importance of the LCS (the number one GCS ranking), the author explored the effectiveness of manipulative and exercise therapy protocols applied alone or in combination for treating CEH. The results showed that both manipulative therapy and specific exercises significantly reduced the frequency and intensity of headaches, while neck pain and associated symptoms remained unchanged. Although the combined treatment was not significantly better than either treatment, 10% of patients achieved relief with the combined treatment. These findings support the use of a combination of manipulative therapy and exercise as a potential treatment option for CEH (31).

The fifth-ranked article in the LCS (GCS = 10) presents a comprehensive review of current knowledge and clinical evidence on CEH. The article examines the fundamental science behind CEH and addresses its recognition, diagnosis, and treatment. At the cervical trigeminal nerve center, a combination of neck and facial nerve signals causes pain in the cervical spine with CEH (32). The article provides evidence for the most effective treatments for CEH. Arthrofusion is the only effective treatment for pain arising from the lateral atlantoaxial joint. The only specific cure for pain from the C2-3 joint is a nerve-blocking procedure using radiofrequency energy (33). The review article provides an exhaustive analysis of current knowledge and clinical evidence about CEH, offering invaluable insights to researchers, clinicians, and healthcare professionals in this domain. The LCS determined that the sixth most impactful study failed to make it to the top 10 list based on the GCS. This study investigated the underlying causes of upper cervical joint dysfunction and revealed that the C1/2 segment and the pectoralis minor muscle length were the primary contributors. Furthermore, compared to the control group, the study identified significant differences in upper cervical joint dysfunction between patients with CEH and those with migraines with Aura (34).

The papers ranked seventh and eighth by the LCS did not feature among the top 10 as determined by the GCS. A previous study investigated the response of patients diagnosed with CEH, migraine without aura, and tension headache to greater occipital nerve block (GON) and supra-orbital nerve block (SN). The results showed that pain reduction after occipital nerve block was more substantial in the CEH group compared to the other patient categories. The study also revealed that relief from forehead pain was mainly observed in CEH patients (35). Another paper investigated the characteristics of CEH in terms of prevalence and clinical indications in the general population. The study found that the prevalence of CEH was 4.1%, with no significant female predominance (F/M ratio of 0.71). The onset of pain in the neck/occipital region and its potential spread to the face were critical features of CEH, with neck pain being a typical symptom of the condition (36).

The ninth most influential paper, as ranked by the LCS, was not in the top 10 according to the GCS ranking. The study investigated the potential susceptibility of small C2/C3 joints to neck trauma. The results showed that in instances where conventional GON blocks were ineffective in reducing pain, C2/C3 synovial joint injections and C3 nerve blocks could significantly alleviate pain and offer additional therapeutic options (37). According to the LCS, the tenth most highly ranked paper did not appear in the top 10 list based on the GCS. This study aimed to determine the prevalence of CEH in the general population and individuals with frequent headaches using a questionnaire-based approach. The findings suggest that CEH, similar to migraine, is a prevalent form of headache in the population (38).

The second most impactful article within the GCS is a clinical guideline that details a systematic overview of the classification, outcome metrics, and therapeutic interventions for musculoskeletal disorders affecting the neck (39). Despite not appearing in the top 10 list of the LCS, the paper holds considerable significance. The literature, which did not feature in the top 10 list of the LCS according to the fourth ranking of the GCS, reinforces the empirical observations made in patients diagnosed with CEH. Patients with this condition commonly display a prominent forward head position and a lack of strength and endurance in the upper cervical flexor muscles. These findings emphasize the importance of rehabilitation targeting endurance and head posture in the clinical management and prevention of CEH (40).

As ranked by the GCS, the fifth most influential paper did not make the LCS top 10 list. Its results suggest that the effects of GON injections on primary headache treatment are not immediate but occur through changes in pain processing pathways and brain plasticity. These findings are crucial for additional research into the different primary headaches (41). The LCS top 10 list did not include the sixth and the eighth most influential papers, as the GCS ranked. The former study confirmed the efficacy of utilizing craniocervical flexion maneuvers as a therapeutic intervention for individuals with neck pain disorders (42). At the same time, the latter investigation revealed the impact of TAMPA scores on functional alterations in the motor system following a neck injury (43).

The literature ranked ninth in the GCS is not included in the LCS’s top 10 list. The paper, a thorough systematic review, examines the benefits of combining manual therapy with exercise for reducing symptoms of neck pain and whether problems in the neck’s nerve accompany it. The review results demonstrate that manual therapy and exercise provide a superior short-term reduction in pain compared to exercise alone. However, the review finds no significant disparities in the long-term results for patients with (sub) acute or chronic neck pain, with or without cervical radiculopathy (44). A study has revealed that individuals who present with neck headaches demonstrate significantly reduced performance in craniocervical flexion tests, specifically in the deep cervical flexor function. The researchers evaluated neck muscles and bones and discovered this finding. Moreover, the researchers discovered a significant association between the deep cervical flexors and the joints of the upper cervical spine. Notably, this finding ranked tenth in the GCS yet was not included in the top 10 list of the LCS (45).

Except for the fourth most impactful paper, as determined by the LCS, which analyzed clinical treatments, all the top documents emphasized the investigation of the diagnostic mechanisms of CEH. According to the GCS, a limited number of three out of the 10 most influential papers overlapped with those listed in the LCS, while the remaining studies centered on the examination of the neck musculoskeletal or comprehensive evaluations of treatments. In addition, research on pain disorders and patient experience is critical to understanding the mechanisms for diagnosing and treating CEH. For example, a study on neck pain by Falsiroli Maistrello et al. (46) highlighted the importance of considering the individual patient’s neck pain experience. Another study by Rossettini et al. (47) emphasized assessing patient satisfaction with physical therapy procedures for musculoskeletal pain conditions.

In addition, there is a need for a comprehensive evaluation of treatment approaches to determine the most effective interventions for managing CEH. A recent study by Rondoni et al. (48) compared active neck range of motion (ACROM) measurements obtained using a technological device with those assessed using a low-cost device for patients with intra- and inter-assessor reliability and showed that there was no significant difference between the technical device and the low-cost device in terms of reliability. Another study by Viceconti et al. (49) investigated the relationship between pain and body perception in musculoskeletal and rheumatic disorders. The results of this study suggest that interventions targeting body perception may be beneficial in improving pain management and quality of life in patients with these diseases.

In conclusion, future research on CEH should focus on examining pain disorders, assessing patient experience, and making a comprehensive assessment of treatment to improve the management of this disease. By incorporating these factors into clinical practice, we can provide more effective treatment for patients with CEH.

### 3.7. Analysis of prominent research trends

**Figure 8:**
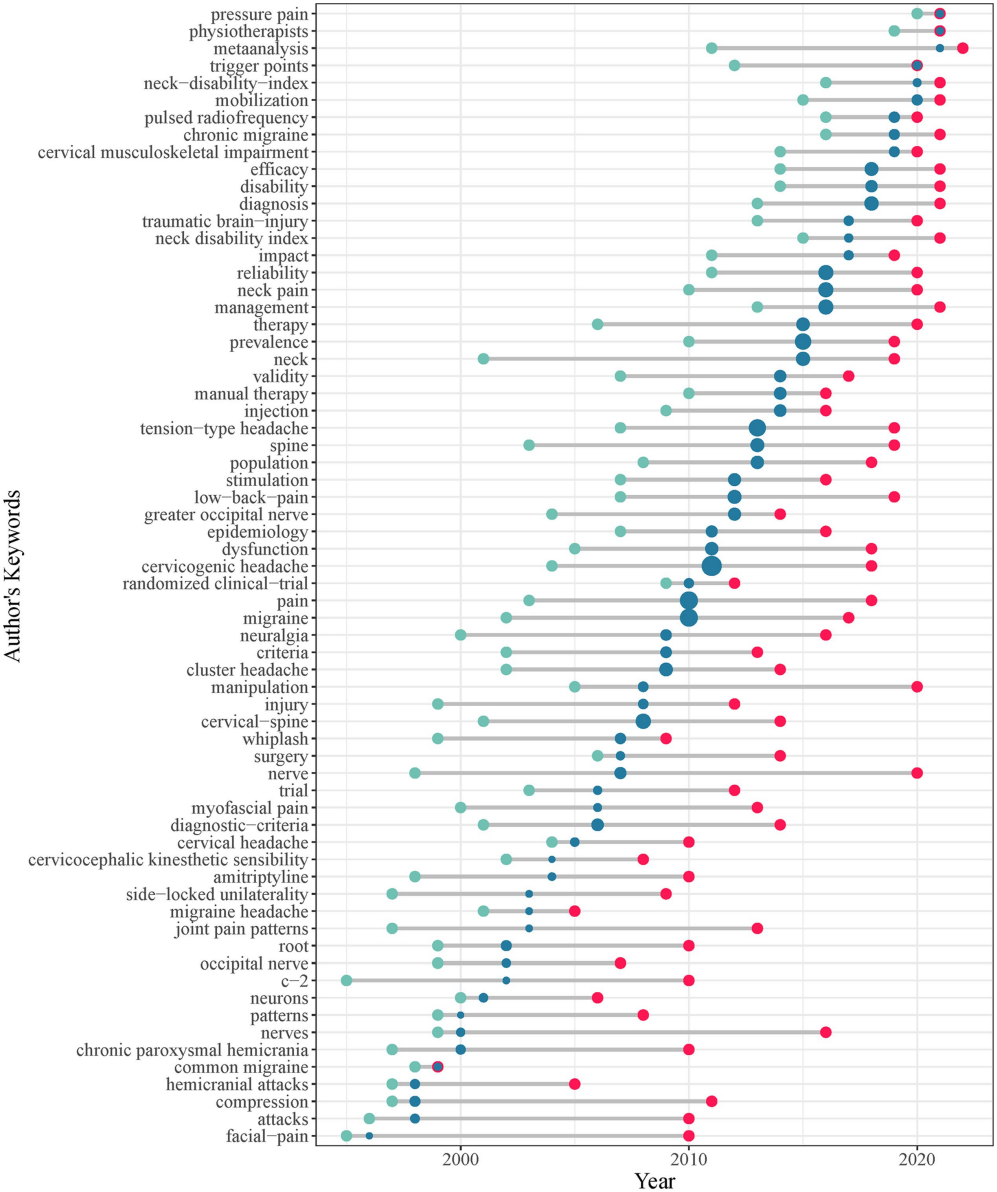
Temporal trends of the author’s keywords.

This study comprehensively analyzed 866 CEH research papers published between 1982 and 2022. Our analysis revealed 1,499 author keywords. Figure 8 presents the author keywords trends over time, showing the publication year on the x-axis and the author keywords on the y-axis. To highlight the trend of research focus, we employed three quantiles (first, median, and third) of the year of publication corresponding to each keyword. The location of the green dot shows the first quantile, the red dot shows the third quantile, and the blue dot shows the median year of publication. The size of each dot indicates the number of papers corresponding to the keyword, with larger dots indicating a higher frequency of keyword occurrences. Two of the most regularly researched topics are “nerve” (50–52) and “neck” (53–55).

Additionally, “spine” (56–58) is another crucial research area that has received considerable attention in CEH research. The frequency of occurrence of keywords in CEH research is represented by blue dots in the center, with larger dots indicating a higher frequency. The top 10 keywords in the field of CEH research include “cervicogenic headache,” “pain,” “migraine,” “tension-type headache,” “prevalence,” “cervical-spine,” “neck pain,” “reliability,” “management,” and “diagnosis.” The most common keyword in CEH research was ‘cervicogenic headache.’ At the same time, ‘pain’ was also a significant area of study, consistent with previous research. “migraine” and “tension-type headache” are two well-researched headache disorders in the field of CEH and are closely related to its studies. Regarding research content, the keywords “cervical-spine” and “neck pain” are primarily used in orthopedic spinal operations (59) and studies investigating the prevalence of these conditions (60). Further significant research directions in the field of CEH include “prevalence,” “reliability,” “management,” and “diagnosis.”

Figure 8 illustrates a relationship between the position of red dots on the right-hand side and the magnitude of the blue dots. Additionally, the figure includes information on the release time of corresponding keywords in the CEH research field. According to the findings, the more recent the publication time, the larger the blue dot and the farther to the right the red dot. The trend reflects the research dynamics in CEH and underscores the significance of manual therapy and randomized clinical trials as critical topics. The recent hot topics in CEH research are “manual therapy” and “randomized clinical-trial.” In clinical practice, manual therapy is widely used to alleviate musculoskeletal pain (61–63).

On the other hand, “randomized clinical-trial” are widely recognized for their superior reliability and rigor in the research process. However, the quality of reporting in randomized controlled trials (RCTs) remains suboptimal (64). In applying CEH clinical studies, “randomized clinical-trial” is mainly used in research investigating manual therapy (65–67).

The current study has uncovered trends in CEH research. The findings indicate a marked improvement in the quality of the diagnosis and treatment research of CEH and a growing diversity in research methodologies. Initially, CEH research focused primarily on “migraine” and “tension-type headache” studies. Subsequently, as the understanding of the research mechanism deepened and methodologies evolved, the sample size expanded. CEH is differentiated from other diseases, and diagnosis and treatment are becoming more accurate. The methodology of CEH research has also evolved. Initially, the research relied on questionnaire surveys and clinical case studies. With an increased prevalence of CEH, current research methods, such as RCTs and meta-analyses, have been incorporated.

### 3.8. Limitations

We conducted a comprehensive literature search of the WOS database, using topics relevant to our research questions. We limited our search to studies published in English from 1982 to 2022. Our search strategy may have introduced some bias because we included only studies published in English and may have missed studies published in other languages. In addition, our search strategy only included WOS databases, which may have excluded studies published in other formats or databases. However, our search strategy was rigorous, and we selected the most appropriate databases for bibliometric studies to minimize bias. In addition, although studies were selected to minimize bias, the quality and risk of bias were not analyzed for the papers in section 3.6. This study covers a wide period to identify trends and changes in the field, but it also means that newer publications are more frequently cited than older ones. The authors are not well-represented in bibliometric studies. Nevertheless, the findings of this study provide valuable insights.

### 3.9. Critical points

In conducting bibliometric analyses, several critical points must be considered to ensure the study’s accuracy. One of the crucial factors is the selection of appropriate keywords to use in the search query. Using good search terms may lead to excluding relevant studies, thus affecting the analysis results. It is also essential to select an appropriate time frame for the search, as this can influence the number and type of publications included in the analysis.

Another critical factor is the selection of appropriate databases to use in the search. While the use of multiple databases can increase the comprehensiveness of the search, it can also result in the inclusion of duplicate or irrelevant studies, which can affect the accuracy of the analysis. Additionally, the quality of the data sources used in the analysis can also impact its validity, making it essential to consider the reliability and validity of the data sources included.

In summary, by addressing these critical points, we can ensure that bibliometric studies provide a more accurate picture of research developments and trends in CEH.

## 4. Conclusion

The field of CEH has experienced a growth in publications in recent years. A bibliometric analysis was conducted to examine the developments and trends in CEH research from 1982 to 2022. The study found that the United States, Australia, Norway, Canada, and Germany contributed significantly to CEH research. The University of Queensland and McMaster University were identified as the primary research institutions. Sjaastad O., Myneni R. B., and Jull G. were the leading researchers in the field. The quality of research data sources has improved, and research methodologies have become more diverse. There has been a broadening of research areas, and the methodology of CEH research has evolved. Multidisciplinary integration is expected to be a significant trend in future research in CEH. Advanced data analysis methods and patient-friendly treatment experience will improve diagnoses and treatment accuracy.

## Data availability statement

The original contributions presented in the study are included in the article/supplementary material, further inquiries can be directed to the corresponding author.

## Author contributions

YX and YD: conceptualization and methodology. LW: data collection. YG and LJ: data curation. YX and YG: formal analysis. YD: funding acquisition and supervision. JY: software. ZY and YD: validation. YX: visualization and writing—original draft. YX, YG, LJ, LW, JY, ZY, and YD: writing—review and editing. All authors contributed to the article and approved the submitted version.

## Funding

This study was supported by the Young and Middle-aged Academic and Technical Leaders Reserve Talents Project of Yunnan Province (grant no. 202105AC160052).

## Conflict of interest

The authors declare that the research was conducted in the absence of any commercial or financial relationships that could be construed as a potential conflict of interest.

## Publisher’s note

All claims expressed in this article are solely those of the authors and do not necessarily represent those of their affiliated organizations, or those of the publisher, the editors and the reviewers. Any product that may be evaluated in this article, or claim that may be made by its manufacturer, is not guaranteed or endorsed by the publisher.
